# Graphene Oxide Mediated Broad-Spectrum Antibacterial Based on Bimodal Action of Photodynamic and Photothermal Effects

**DOI:** 10.3389/fmicb.2019.02995

**Published:** 2020-01-15

**Authors:** María Paulina Romero, Valeria Spolon Marangoni, Clara Gonçalves de Faria, Ilaiali Souza Leite, Cecília de Carvalho Castro e Silva, Camila Marchetti Maroneze, Marcelo A. Pereira-da-Silva, Vanderlei Salvador Bagnato, Natalia Mayumi Inada

**Affiliations:** ^1^São Carlos Institute of Physics, University of São Paulo, São Carlos, Brazil; ^2^Department of Materials, Escuela Politécnica Nacional, Quito, Ecuador; ^3^MackGraphe, Mackenzie Presbyterian University, São Paulo, Brazil

**Keywords:** photodynamic-photothermic effect, singlet oxygen, broad-spectrum antibacterial, graphene oxide, nano graphene oxide, bacterial decontamination

## Abstract

Graphene oxide (GO) with their interesting properties including thermal and electrical conductivity and antibacterial characteristics have many promising applications in medicine. The prevalence of resistant bacteria is considered a public health problem worldwide, herein, GO has been used as a broad spectrum selective antibacterial agent based on the photothermal therapy (PTT)/photodynamic therapy (PDT) effect. The preparation, characterization, determination of photophysical properties of two different sizes of GO is described. *In vitro* light dose and concentration-dependent studies were performed using Gram-negative *Escherichia coli* and Gram-positive *Staphylococcus aureus* bacteria based on the PTT/PDT effect used ultra-low doses (65 mW cm^–2^) of 630 nm light, to achieve efficient bacterial decontamination. The results show that GO and nanographene oxide (nGO) can sensitize the formation of ^1^O_2_ and allow a temperature rise of 55°C to 60°C together nGO and GO to exert combined PTT/PDT effect in the disinfection of gram-positive *S. aureus* and gram-negative *E. coli* bacteria. A complete elimination of *S. aureus* and *E. coli* bacteria based on GO and nGO is obtained by using a dose of 43–47 J cm^–2^ for high concentration used in this study, and a dose of around 70 J cm^–2^ for low dose of GO and nGO. The presence of high concentrations of GO allows the bacterial population of *S. aureus* and *E. coli* to be more sensitive to the use of PDT/PTT and the efficiency of *S. aureus* and *E. coli* bacteria disinfection in the presence of GO is similar to that of nGO. In human neonatal dermal fibroblast, HDFs, no significant alteration to cell viability was promoted by GO, but in nGO is observed a mild damage in the HDFs cells independent of nGO concentration and light exposure. The unique properties of GO and nGO may be useful for the clinical treatment of disinfection of broad-spectrum antimicrobials. The antibacterial results of PTT and PDT using GO in gram-positive and gram-negative bacteria, using low dose light, allow us to conclude that GO and nGO can be used in dermatologic infections, since the effect on human dermal fibroblasts of this treatment is low compared to the antibacterial effect.

## GRAPHICAL ABSTRACT

PTT/PTT effect due to irradiation with red LED of GO and nGO in the presence of *E. coli* and *S. aureus* bacteria.

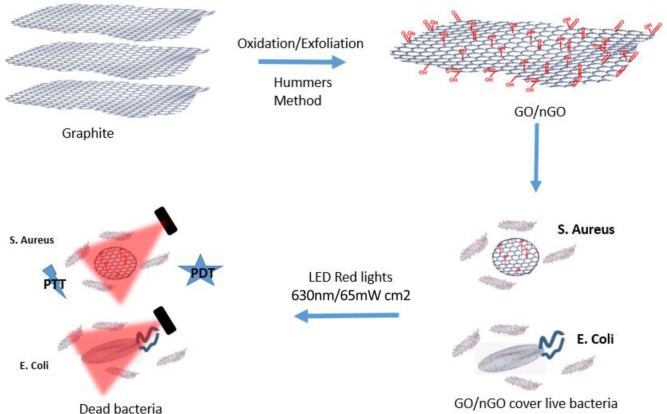

## Background

According to the World Health Organization, there are new mechanisms of microbial resistance that are spreading all over the world. This threatens our ability to treat common infectious diseases; resulting in prolonged illness, disability and death (WHO, 2018). Therapies with conventional antibiotics are becoming less efficient due to the appearance of bacterial strains resistant to them. The development of new antibacterial material to effectively inhibit or kill bacteria is crucial (WHO, 2014). Microbial disinfection approaches that use graphene-based materials [e.g., graphene oxide (GO), nanographene oxide (nGO), and graphene quantum dot (GQD)] are relatively simple, affordable, and adaptable.

The exploration of antimicrobial nanomedicine in current clinical practice is very limited, but it has great potential (Karaham et al., 2018). Such as, Chong et al. (2017) reported how exposure to simulated sunlight significantly enhances the antibacterial activity of GO (Chong et al., 2017). On the other hand, a decrease in the bacteria population have been observed when they come in contact with the GO layer area and its antimicrobial activity increases with deceasing sheet area (Perreault et al., 2015). Jilani et al. (2018) deposited GO thin films of different concentrations on glass substrate by spin coating technique. GO thin films were subjected for antibacterial activity tests toward *Bacillus cereus* and *Serratia marcescens* and significantly inhibit the bacterial growth in both solid and liquid media (Jilani et al., 2018). Kumar et al. (2016) prepared a polyaniline@TiO2/graphene (Pani@TiO2/GN) nanocomposite by the *in situ* oxidative polymerization of aniline in the presence of TiO2 and GN nanoparticles. Pani@TiO2/GN revealed high antibacterial activity toward *Escherichia coli* and Enterobacter ludwigii, highlighting its potential as a photocatalyst with antibacterial properties for different industrial and medical purposes (Kumar et al., 2016). Wang et al. (2018) reported that acrylic acid and N, N’-methylene bisacrylamide were crosslinked with GO hydrogels. With a 5: 1 ratio of Ag to GO, a good biocompatibility, a strong antibacterial capacity and a high swelling ratio with sufficient mechanical resistance were obtained, which led to a greater wound healing ratio during a 15-day observation (Wang et al., 2018). Furthermore, recently GO-metal hybrid antimicrobial activity was observed against medically important microbes *E. coli*, *Staphylococcus aureus*, *Enterococcus faecium*, and *Klebsiella pneumonia* (Whitehead et al., 2017).

Graphenes are emerging members of the carbon family with great potential due to their extraordinary thermal and optoelectronic properties. Their two-dimensional structure, large surface area, easy surface functionality, biocompatibility, high thermal stability, low cost in comparison to other nanoparticles, and their NIR absorption capacity in the first and second biological windows (650–950 nm) and (1000–1350 nm), make them ideal for diagnosis and treatment with photodynamic therapy (PDT) and photothermal therapy (PTT) (Robinson et al., 2011). GO could also induce oxidative stress when in direct contact without the participation of ROS (Liu et al., 2011). GO sheets can oxidize glutathione effectively (Liu et al., 2011).

Photothermal therapy is a minimally invasive, local treatment modality that mainly relies on an optical absorbing agent, also known as photosensitizer, which can absorb energy and convert it into heat upon stimulating by an electromagnetic radiation such as radiofrequency, microwaves, near infrared irradiation, or visible light. The principal effect of PTT include the capability for deep tissue penetration and minimal effect of non-selective cell death on the surrounding healthy tissue, this process is called hyperthermia (Eskijzmir et al., 2018; Fong et al., 2018). Hyperthermia may trigger denaturation of proteins, lysis of cell membrane, evaporation of cytosol, thereby leading to cell death (Roti Roti, 2008). PTT induces programed cell death (apoptosis) by activating the intrinsic pathway rather than necrotic cell death. The necrotic cell death stimulates inflammatory responses which may usually compromise the antitumor activity (Pérez-Hernández et al., 2015). PTT is used for both tumor removal (Yang et al., 2010; Robinson et al., 2011; Tu et al., 2014; Wang et al., 2015) and bacterial disinfection in various types of bacteria (He et al., 2018; Al-Bakri and Mahmoud, 2019; Qing et al., 2019).

Photodynamic therapy involves the administration of a photosensitizer (PS), molecular oxygen and a light source and is used to treat cancer (Castano et al., 2006; Fan et al., 2016) and kill microorganisms for management of infectious diseases (Wainwright et al., 2017). In PDT, cell damage is generated by reactive oxygen species (ROS) such as hydroxyl radical (OH), singlet oxygen (^1^O_2_), and superoxide radical (O2^–^), which are produced by the reaction of the excited PS and oxygen (O_2_). This is a highly localized effect due to the high reactivity and short lifetime of ROS (Dolmans et al., 2003; Lan et al., 2019). PDT exhibits several advantages including non-invasiveness, no drug resistance, low cytotoxicity, selective targeting, spatiotemporal precision, and synergistic effect over conventional therapeutic modalities (Li et al., 2019). Being ^1^O_2_ a primary toxic photochemical product that directly or indirectly destroys tumor cells via apoptotic, necrotic and autophagy-associated cell death that cause cellular apoptosis, are generated (Dougherty et al., 1998; Dou et al., 2016).

Complex formulations based on GO have been studied as new types of broad spectrum antimicrobial agents and have been used for the design of wound dressings, protective coatings against infections and formulations similar to that of antibiotics (“nanoantibiotics”) (Bussy et al., 2013; Szunerits and Boukherrroub, 2016). Xie et al. (2017) reported efficient synergistic destruction of AgNPs-based bacteria, which were uniformly distributed in a well-defined GO sheet through *in situ* reduction of Ag+; his nanocomposite was wrapped in a thin layer of collagen. Ag+ release and ROS generation induced by light was observed. An *in vivo* subcutaneous test showed that irradiation at 660 nm could achieve a highly efficient antibacterial disinfection of 96.3% and 99.4% against *E. coli* and *S. aureus*, respectively (Xie et al., 2017).

Tian et al. (2014) reported on a synergistic and recyclable nanocomposite by growing both iron oxide nanoparticles (IONP) and silver nanoparticles (AgNP) on the surface of Go sheets. The GO-IONP-Ag nanocomposite showed a higher antibacterial efficacy against gram-negative than that shown by gram-positive bacteria. The Photothermal treatment is carried out due to a strong absorbance in the near infrared (NIR) by GO-ION-Ag (Tian et al., 2014). All of them have used complex nanocomposites to obtain the double PTT/PDT effect that induces ROS generation.

In this study, two sizes of GO were used to study the microbiological effects after illumination; and its cellular cytotoxicity was studied using a threshold dose analysis. It was shown that GO and nGO can exhibit wavelength-dependent photon excitation using a simple low-intensity LED array, that induces the generation of ^1^O_2_ and mediates the bimodal effects of PDT and PTT in the destruction of *S. aureus* and *E. coli* bacteria.

## Materials and Methods

### Preparation and Characterization of Graphene Oxide and Nanographene Oxide

Graphene oxide was prepared and characterized following previous reports (Hummers and Offeman, 1958). GO (3 mg/mL) was prepared following a modified Hummers method and using a 7-day oxidation time. A 1.3 mg/mL nGO concentration was obtained from the GO suspension, by heating the GO suspension at 80°C for 24 h in a mixture of sulfuric and nitric acid (4:1 v/v). The resulting solution was neutralized, sonicated for about 3 h at 50°C, and purified by repeated dialysis (MWCO 15 kDa) in ultrapure water.

The shape and thickness of GO and nGO samples were characterized by tapping-mode atomic force microscopy (Bruker Dimension Icon AFM), Dynamic Light Scattering and Zeta Potential (Malvern Zetasizer Nano ZS90). Transmission electron microscopy (TEM) was performed in a JEOL – JEM2100 LaB6 HR operating at 200 kV. Samples for TEM were prepared by drop casting of nGO and GO dispersion on ultra-thin carbon film-coated copper grid, 300 mesh. Luminescence emission measurements were performed at room temperature on a Cary Eclipse, Agilent technology spectrofluorometer and a Varian Cary^®^ 50 UV-VIS system spectrophotometer.

Other techniques, including infrared spectroscopy (Agilent Technologies, Cary 630 FTIR), Raman spectroscopy (Renishaw RM2000, laser HeNe and 632.8 nm wavelength) were adopted to characterize the GO and nGO samples.

### Bacterial Suspension and Treatment

The experimental process of bacteria suspension S. *aureus* and *E. coli* is detailed in Supplementary Material E1. The concentration of GO or nGO in the bacteria suspension (10^8^ Colony Forming Units ml^–1^) is cited in Table 1.

**TABLE 1:** GO and nGO concentrations in *Staphylococcus aureus* and *Escherichia coli* suspensions used in this experiment.

	***S. aureus* (mg ml^–1^)**	***E. coli* (mg ml^–1^)**
GO	0.75	1.5
GO	0.30	1.1
GO	0.15	0.45
nGO	0.595	0.744
nGO	0.446	0.536
nGO	0.179	0.357

The dose-response experiments were designed for photoexcitation in different concentrations of GO and nGO (Table 1) using different fluencies: 0, 40, 60, and 80 J cm^–2^ (630 nm; 65.5 mW cm^–2^) corresponding to irradiation times of 0, 10, 15, and 20 min, respectively. Three replicates were performed for each experimental group.

### Bacteria Imaging by Atomic Force Microscopy

The interaction between the bacteria and GO or nGO was evaluated by atomic force microscopy (AFM). For this, 10 μL of the previous bacterial suspension was deposited directly onto the clean glass slide and dried at room temperature for 10 min (Liu et al., 2012). AFM images were obtained using a Bruker, tapping mode, and silicon tip. Dimension Icon spring constant 40 N/m, with an oscillation frequency of 320 KHz.

### Threshold Dose Analysis

The dose-response curve is a relationship of how an organism responds to different levels of a measurable stimulus (such as the dose of light). Cell death response is measured on a binary scale: response or non-response and the level of stimulus that separates them is called the threshold dose. Since not all subjects in a population have the same tolerance, due to variability between individuals, the dose response curve has a sigmoid shape (Sabino et al., 2011). The threshold dose analysis is detailed in Supplementary Material E2.

### 1,3-Diphenyl Isobenzofuran Quenching Experiments

1,3-diphenyl isobenzofuran (DPBF) is utilized as a singlet oxygen trapping agent with a strong absorption of light, around 410–420 nm and emission of blue fluorescence. DPBF reacts with ^1^O_2_ to form o-dibenzoylbenzene, which does not absorb visible light. The decrease in DPBF absorbance reflects the amount of ^1^O_2_ (Romero et al., 2013) generated.

1,3-diphenyl isobenzofuran and different concentrations of GO and nGO were mixed in Tween 80 solution and irradiated with a 630 nm light (65.5 mW cm^–2^) at different time intervals, and the absorbance at 418 nm was measured using a UV-VIS spectrophotometer. All measurements were performed in air-saturated solutions and temperature controlled light irradiation.

### *In vitro* Cellular Uptake Experiments

Fibroblasts were chosen to assess how GO and nGO-mediated PDT would affect healthy human cells when submitted to the conditions that promoted bacterial inactivation. The experimental process is detail in Supplementary Material E3.

## Results

### Characterization of Graphene Oxide and Nanographene Oxide Morphology

**FIGURE 1:**
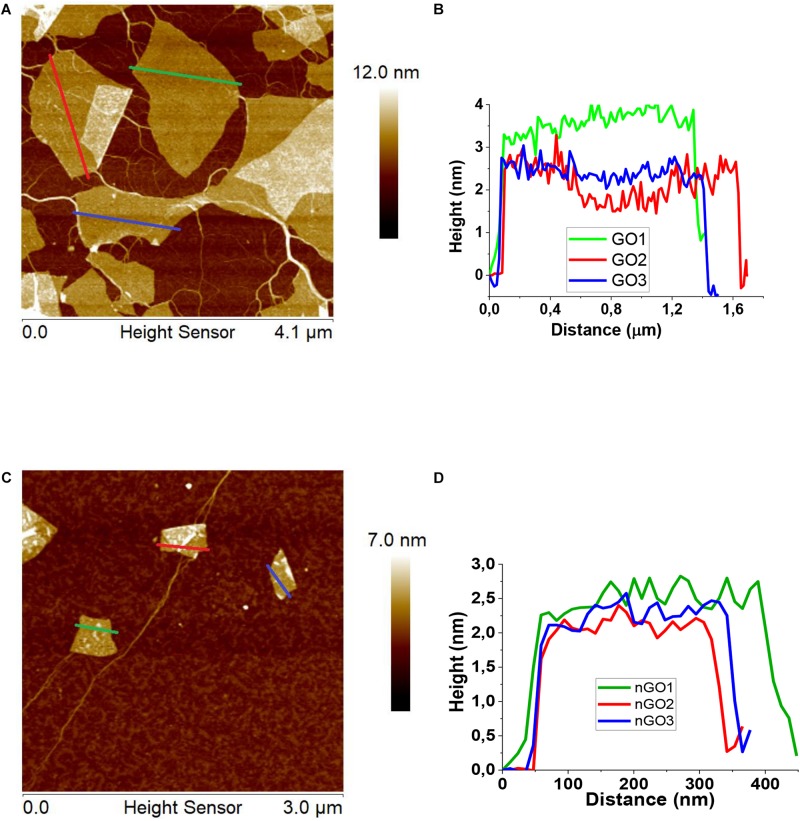
Characterization of GO and nGO. **(A,C)** Atomic force microscopy (AFM) image, **(B,D)** The height profiles along the lines of the panel from GO and nGO, respectively.

Graphene oxide was characterized by AFM. As displayed in Figures 1A,B, the height profile along the small lines indicates that the thickness of the GO is about 2.8 nm, suggesting the sheet is four-layered (Sun et al., 2010). Different GO sheet sizes are shown but the predominant size is about 2 μm. In the case of nGO, the AFM characterization (Figures 1C,D) shows that the thickness of the nGO is about 2.0 nm suggesting the sheet is three-layered (Sun et al., 2010), the different sizes are shown in Figure 1D. The predominant size from distribution is about 0.34 μm.

A TEM image of the nGO sheet is shown in Supplementary Figures S1D–F, show carbon nanoparticles. The corresponding fast Fourier Transform (FFT) pattern reveals that these structures are crystalline with a lattice distance of 0.20 ± 0.01 nm, corresponding to the (100) plane of graphene (Liu et al., 2015). In contrast, TEM images of the GO sheet (Supplementary Figures S1A–C) and amorphous carbon nanoparticles without any discernible lattice fingers are shown.

### Characterization of Graphene Oxide and Nanographene Oxide Composition and Structure

A fourier transform infrared spectroscopy (FTIR) was performed on these samples in order to study the difference in oxygen-related functional groups in GO and nGO (Supplementary Figure S2). GO shows peaks corresponding to the frequencies of -OH (3200 cm^–1^), C-H (2928 and 2850 cm^–1^), COOH (1721 cm^–1^), C = 0 (1615 cm^–1^), C-O (1225 cm^–1^), C-OH (1056 cm^–1^), and C-O-C (850 cm^–1^) (Sarkar et al., 2016; Tang et al., 2016). In comparison with GO, the FTIR spectra of nGO reveals the mostly absent absorption band of C-O-C, the relative intensity of C = O, and the decreased absorption band of C-OH. Those spectra feature suggest that an nGO is obtained via cutting C-O-C groups of GO into C-O. The variance of oxygen content in GO and nGO functional groups indicates their difference in structure. A Raman spectroscopy was performed to further confirm this fact (Supplementary Figure S3). In the Raman result, the D-band (related to sp^3^ hybridized carbon atoms and to the presence of structural defects) and the G-band (associated with sp^2^ hybridized carbon atoms) are bands characteristic to carbon-based material. The I_D_/I_G_ ratio can be used to estimate the crystalline quality of the material. For GO and nGO, the peaks around 1340 cm^–1^ and 1597 cm^–1^ correspond to D and G bands. The I_D_/I_G_ ratios for GO and nGO are calculated to be 1.19 and 0.97, respectively. These results indicate that the nGO shows a higher level of crystallinity compared to that of GO and agrees with the distinct lattice fringe image (Supplementary Figure S1F).

### Graphene Oxide and Nanographene Oxide Optical Properties

**FIGURE 2:**
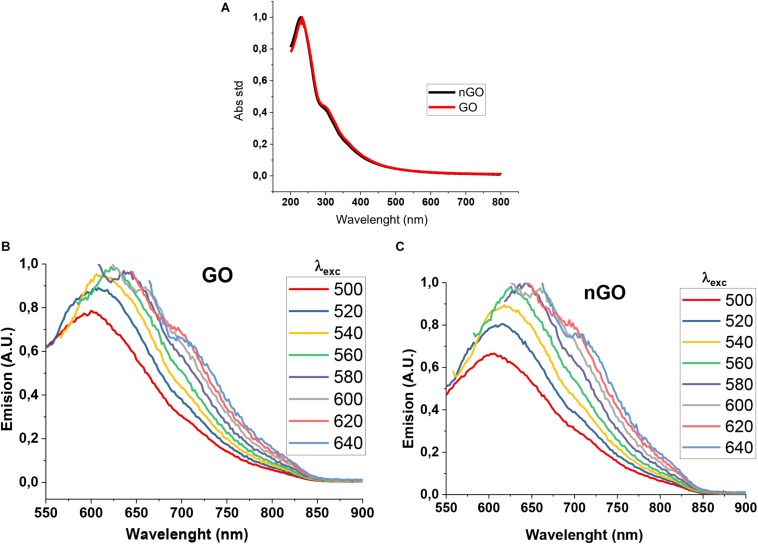
Optical properties of GO and nGO with different concentrations. **(A)** UV-VIS spectra. **(B,C)** Emission spectra of GO and nGO, respectively.

UV-VIS absorbance spectra and photoluminescence emission were obtained. Figure 2A, indicates that GO and nGO have an absorption band at λ < 240 nm (characteristic of the π-π^∗^ transitions of aromatic bonds C = C), and another smaller band around 300 nm which belongs to the n-π^∗^ transition of C = O or the COOH group (Liu and Kim, 2015; Tang et al., 2016). There is no considerable difference in the absorbance spectrum between GO and nGO. The photoluminescence spectra of GO (Figure 2B) and nGO (Figure 2C) were obtained at different excitations, Jin et al. (2012) observed that under certain critical limit sizes, GO can show permanent photoluminescence, arising from quantum confinement effects. Both samples display fluorescence under several wavelength irradiation (Jin et al., 2012). The red shift in both samples is present, depending on the excitation.

### Atomic Force Microscopy Images of Bacteria

**FIGURE 3:**
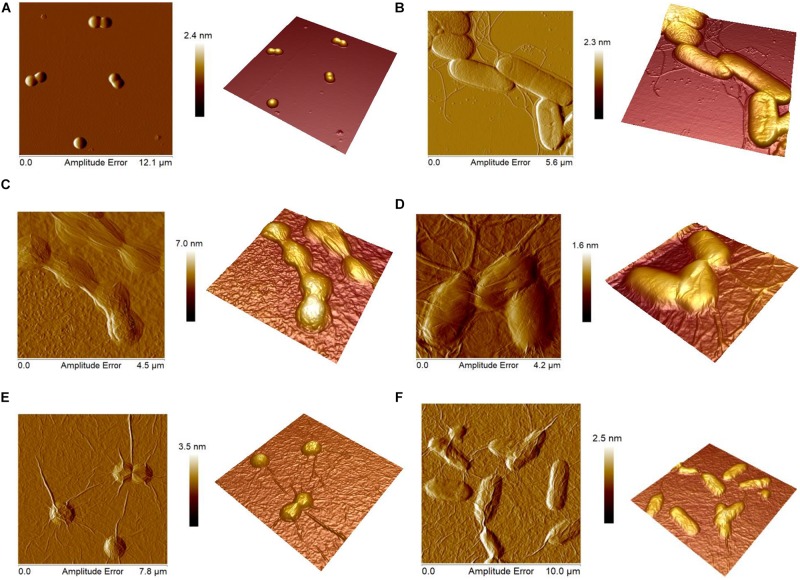
Atomic force microscopy images showing a 2D amplitude image at left and height 3D image at right. **(A)** *Staphylococcus aureus*. **(B)** *Escherichia coli*. **(C)** *S. aureus*-GO (300 μg ml^–1^). **(D)** *E. coli*-GO (1.13 mg ml^–1^). **(E)** *S. aureus*-nGO (446.3 μg ml^–1^). **(F)** *E. coli*-nGO (535.5 μg ml^–1^). All solutions were incubated with deionized water for 45 min threshold dose analyses for phototoxicity.

Atomic force microscopy images of GO and nGO systems in *S. aureus* and *E. coli* solution were obtained. Figures 3A,B show AFM images of *S. aureus* and *E. coli* without GO or nGO, where a smooth surface of bacterial cell walls is observed. In GO-*S. aureus* and GO-*E. coli* images (Figures 3C,D) it is possible to observe that bacteria were covered by large sheets of GO, similar to that of nGO-*S. aureus* and nGO-*E. coli* (Figures 3E,F).

Taking into account the concentration levels of GO and nGO, it can be seen that GO has twice the concentration of nGO; therefore, it is necessary to carry out a thorough analysis of its effect on the results obtained. An analysis of surface roughness, will allow a study of GO and nGO adhesion on bacteria *S. aureus* and *E. coli*. The surface roughness value of *S. aureus*, *E. coli*, GO-*S. aureus*, GO-*E. coli*, nGO-*S. aureus*, nGO-*E. coli* were obtained and shown in Table 2. After the incubation of *S. aureus* and *E. coli* with GO and nGO, the cell surface roughness increased in relation to the solution containing only bacteria.

**TABLE 2:** Data roughness, concentration and Dp of the GO and nGO in the presence of *S. aureus* and *E. coli*.

	**Roughness (nm)**	**Concentration GO and nGO (μg ml^–1^)**
*S. aureus*		
Only bacteria	0.76 ± 0.30	
Plus GO	3.22 ± 0.68	300
Plus nGO	2.20 ± 0.45	446.5
*E. coli*		
Only bacteria	1.32 ± 0.25	
Plus GO	1.45 ± 0.26	1130
Plus nGO	1.78 ± 0.97	535.5

A greater concentration of GO and nGO present in the solution allows a complete coating of *E. coli* but decreases the roughness (Table 2). A greater roughness value is observed for the *S. aureus*-GO and *S. aureus*-nGO systems. Taking into account that the concentration of GO and nGO in the presence of *S. aureus* is the lowest in relation to all the systems studied, we can conclude that due to the size of the *S. aureus* bacteria (1 μm), it requires low concentrations of GO and nGO to be completely coated to the point of showing a high surface roughness in relation to the control of *S. aureus*. Due to its size (3 μm), *E. coli*, requires a higher concentration of GO and nGO to be completely covered without significantly influencing the increase in surface roughness in relation to control E. *coli.*

In this work, the bacterial toxicity mechanism based on PDT- PTT therapies needs to be clarified for their various potential applications. No significant aggregation or solid particle precipitation was observed during the incubation carried out during all the tests.

**FIGURE 4:**
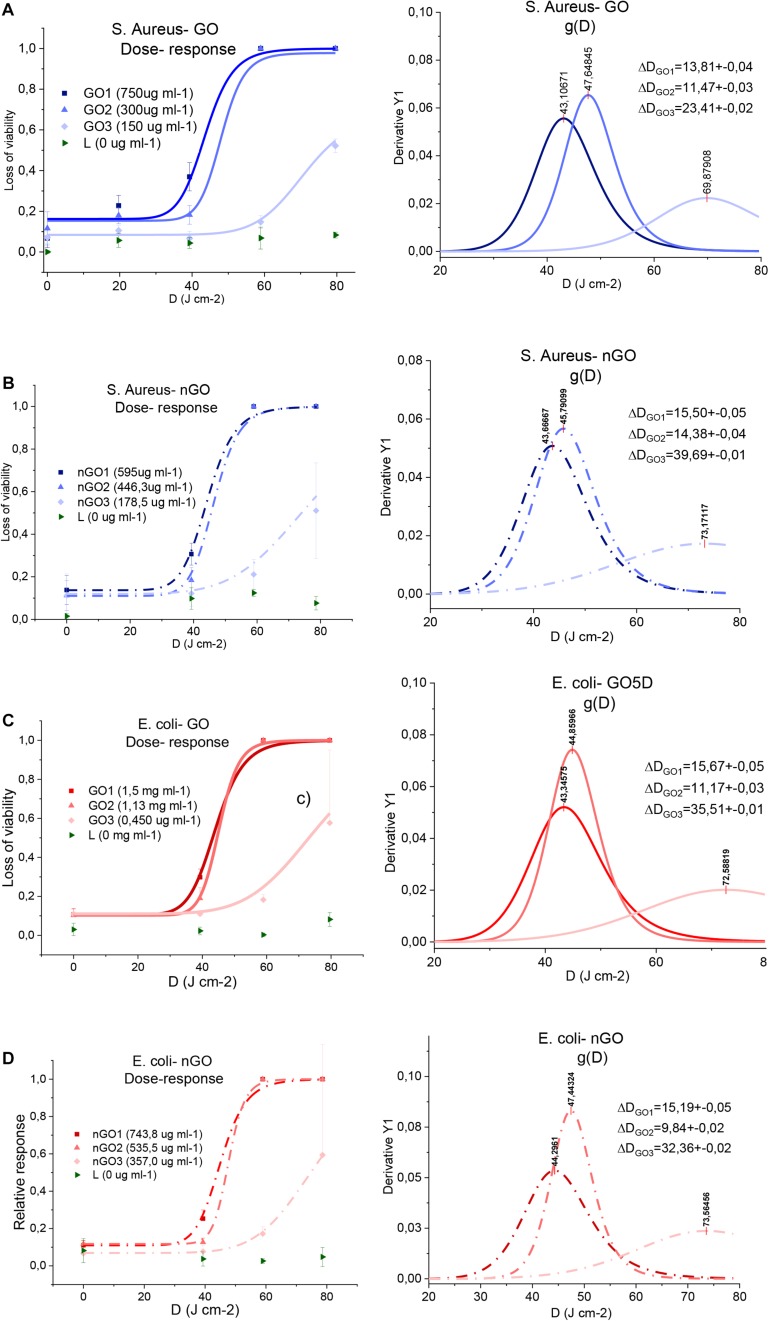
Dose-response curves and the threshold dose distribution. **(A)** *S. aureus*-GO, **(B)** *S. aureus*-nGO, **(C)** *E. coli*-GO, and **(D)** *E. coli*-nGO.

Dose-response curves for PDT experiments and their corresponding threshold dose distributions are shown in Figure 4. These curves were obtained from the result of colony counting obtained by irradiation of *S. aureus* and *E. coli* bacteria in the presence of GO and nGO as a direct killing effect.

**FIGURE 5:**
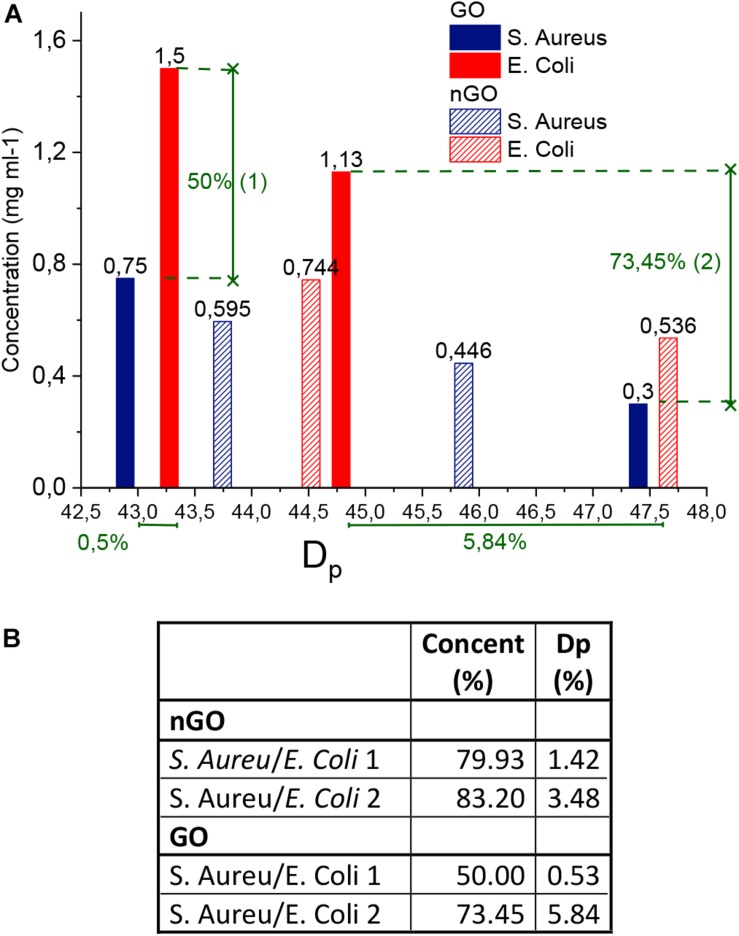
Threshold dose parameters. **(A)** *D*p. **(B)** Ratio of concentrations (%) and reduction percentage in ratios of GO and nGO for *S. aureus* and *E. coli* [(1-ratio)^∗^100%].

Parameter *D*_P_ is summarized in Figure 5 and is shown as a function of GO and nGO concentrations. In order to continue with this analysis, the low GO and nGO concentration curves obtained were eliminated.

This due to the fact that they do not appear complete in the dose-response curves (below 60% of cell death), and so it is not certain that the *D*_P_ value in the threshold dose curve is accurate. It is noticeable that all *D*_P_ values are dependent on the concentration of GO or nGO: larger nGO and GO concentrations imply lower *D*_P_ values. Low *D*_P_ values mean that a low light dose is required to eliminate a large population fraction, while high ones are due to poor response in conditions of low concentrations.

In this study, the highest value of *D*_P_ corresponds to the GO- *S. aureus* system, followed by nGO- *E. coli*; indicating that these are the most resistant conditions tested. *S. aureus* and *E. coli* have very similar sensitivities in the presence of the highest concentration of GO. Surprisingly, the most sensitive bacteria for low GO or nGO concentrations is different, *S. aureus* is more resistant in the lowest GO concentration levels while *E. coli* is so for the low nGO system.

An analysis of the reduction percentage in the ratio between two *D*_Ps,_ is defined as: [1- *D*_P__1_/*D*_P__2_)^∗^100%], in terms of GO, and nGO concentration and is summarized in Figure 5B. An example of it is also indicated in the graph for a clearer understanding. Comparisons were made between high (WHO, 2018) and low (WHO, 2014) concentrations of each of the systems and it highlights the influence of GO and nGO concentration in bacteria response to PTT/PDT. When comparing bacteria types, the only significant differences in *D*_P_ between *S. aureus* and *E. coli* are for low concentration situations: 3.48% for nGO and 5.84% for GO. This means that at higher concentrations the differences in sensitivity of the different species are insignificant. From the point of view of graphene systems, it can be observed that in general the graphene conditions tested (whether in the intervals of low or high concentrations), are not proportional to the changes in *D*_P_ when they increase. For the *S. aureus*-GO system, for example, the concentration was doubled for a reduction of 9.53% in *D*_*P.*_ Nevertheless, nGO showed a better response to changes in concentration compared to GO, especially for *E. coli*; where a similar increase in concentration yield a in double the reduction fraction.

**FIGURE 6:**
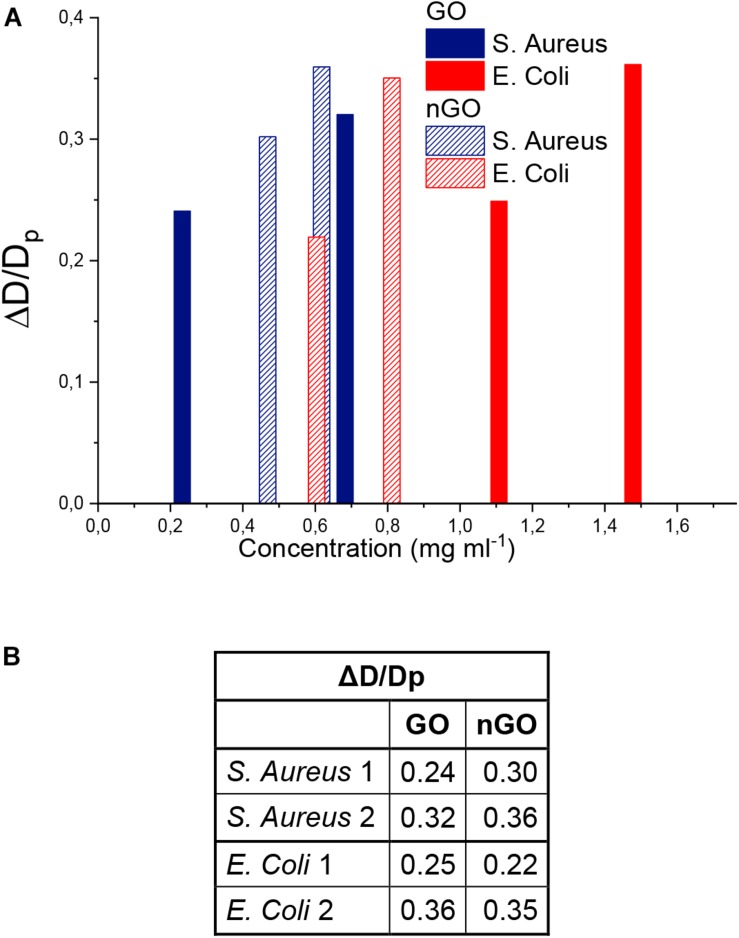
ΔD/DP ratio. **(A)** All systems. **(B)** Box ΔD/DP summarized dates.

Parameter Δ*D* is used to analyze how homogeneous the systems response is. The relative variability, given by the ratio Δ*D/D_P_* (Figure 6) is shown to account for the intrinsic distribution broadening behavior as *D*_P_ increases. It is observed that it increases with concentration, therefore, low quantities of GO and nGO yield a more homogenous response, since variability is minimum among the conditions tested. In GO-based systems, *S. aureus* and *E. coli* present similar values for lower and higher concentration, while nGO-*E. coli* Δ*D/D_P_* values are lower in relation to the ones for *S. aureus*.

**FIGURE 7:**
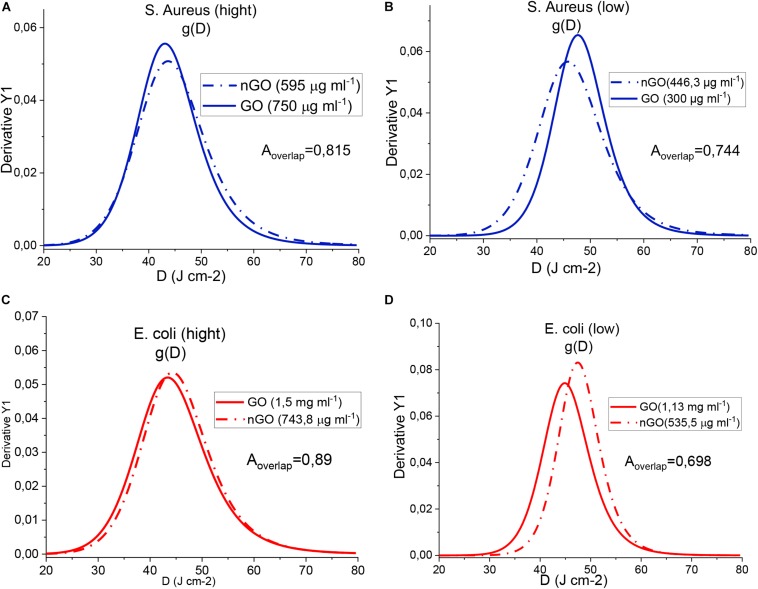
Area of overlap between the threshold dose distributions of the systems. **(A)** *S. aureus* (high). **(B)** *S. aureus* (low). **(C)** *E. coli* (high). **(D)** *E. coli* (low).

The overlap area of threshold dose distributions for each bacterium is shown in Figure 7. It represents the fraction of the population that would be destroyed via PTT/PDT in both GO and nGO conditions (de Faria et al., 2018). So, the closer to 1 the area is, the more similar the conditions are. Therefore, it is concluded that nGO is more efficient since the overlap is greater than 70% for all the conditions tested at lower concentrations than GO. For *S. aureus*-low concentrations (GO and nGO) nGO were found to be better, since the distribution is shifted toward the left. This indicates that the population is more sensitive to that condition. *E. coli*-low is the least favorable result, as it still includes 30% of the population that responds to GO but is not killed with nGO at 535.5 μg/ml.

The efficiency of *S. aureus* and *E. coli* bacteria disinfection in the presence of GO is similar to that of nGO. The presence of high concentrations of GO allows the bacterial population of *S. aureus* and *E. coli* to be more sensitive to the use of PTT/PDT. The results obtained in this study suggest that the use of GO and nGO for the disinfection of *S. aureus* and *E. coli* bacteria by PTT/PDT is efficient. These results obtained with a PTT/PDT cycle should continue to be investigated with more cycles of PTT/PDT to answer questions such as the virulence factor of bacteria in the presence of GO and nGO, and the efficiency of the treatment through several cycles of PTT/PDT with a single dose of GO and nGO in the near future.

### Photodynamic Analyses

Quenching of DPBF in the presence of GO and nGO was used in order to evaluate whether GO or nGO mediated PDT effects can achieve effective antibacterial action of *S. aureus* and *E. coli* using 630 nm light 65.5 mW cm^–2^. The absorbance spectrum of DPBF + GO system is a superposition of the GO and DPBF spectra (Supplementary Figure S4), in different time irradiations the DPBF band (418 nm) decreases.

**FIGURE 8:**
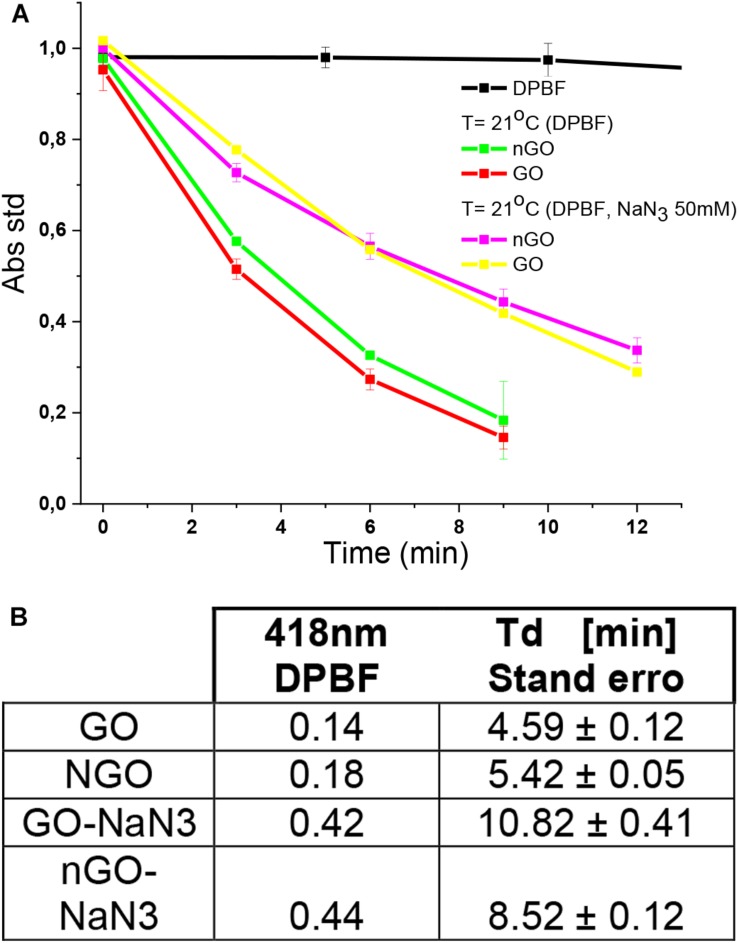
**(A)** Decay curve of DPBF at 21°C. **(B)** Time decay of DPBF from GO and nGO. DPBF = 5 μm, GO = 30 μg ml^–1^, nGO = 31.7μg ml^–1^, and NaN3 = 50 mM.

Different concentrations of GO and nGO quenching the DPBF upon irradiation using 630 nm light is shown in Supplementary Figure S5. The graphs for each concentration of GO and nGO were fitted with an exponential decay equation, whose decay time is summarized in Supplementary Figure S6. As expected, when increasing the concentration of GO and nGO; a decrease in the time of decay of the absorption intensity at the 418 nm band of DPBF was observed. It is observed that for similar concentrations of GO and nGO, the decay time constants are similar. The generation of ^1^O_2_ by the photoexcitation of GO and nGO was confirmed by DPBF quenching studies, where the optical absorbance of DPBF at 418 nm decays continuously upon 630 nm irradiation of the solution in the presence of GO and nGO. In the absence of GO and nGO, the DPBF solution does not exhibit any noticeable decay in absorbance. A new photooxidation experiment of DPBF was performed in the presence of GO, 24 h after the first irradiation and a total photo degradation. A time delay of DPBF band similar to that of the first experiment was observed for the new concentration of DPBF, in the presence of GO. This can be explained due to the better dispersion of GO layers in the presence of surfactant Tween80; and this allows a better distribution of DPBF between the GO layers. The same behavior in the interaction nGO-DPBF is expected because nGO has the same GO structure with only a small difference in its oxygen content (FTIR image Supplementary Figure S2). A sodium azide pretreatment was performed (Kalluru et al., 2016) in order to determine whether ^1^O_2_ is the key ROS or not. The result in Figure 8 clearly shows that ROS levels were drastically suppressed in DPBF irradiated with 630 nm light. Therefore, the results point out that the quenching of DPBF is mostly due to GO and nGO mediated PDT effects.

### Thermal Treatment

The exposure of GO and nGO to 630 nm red LEDs, induce the conversion of optical energy into thermal energy, rapidly generating localized heat that causes the thermal ablation of bacteria.

**FIGURE 9:**
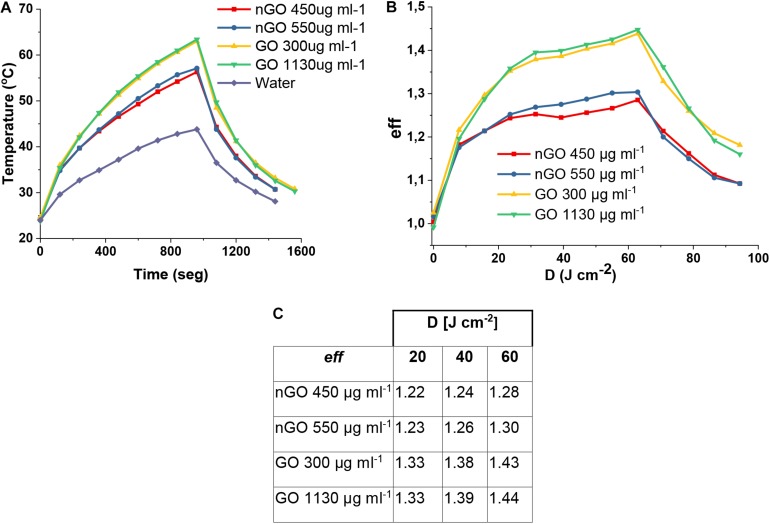
**(A)** Average temperature of GO and nGO. **(B)** PTT heating efficiency (eff). **(C)** Box summary of eff.

The photothermal temperature measurement for the aqueous solutions of GO and nGO, with concentrations shown in Table 1; upon photoexcitation using 630 nm lights (Figure 9A), clearly exhibit a change in temperature for nGO and GO solutions. As can be seen in Figure 9A, the temperature achieved when irradiating with 630 nm, 65 mW cm^–2^ light for about 16 min (1000 s) for the two concentrations of nGO used in this study, was around 55°C. For GO, the temperature achieved in the two concentrations is around 63°C. Kim et al. (2013) observed that a high local temperature must be achieved to thermally inactivate *S. aureus* because they have a higher thermal tolerance than cancer cells. The local temperature of 60–70°C, depending on the duration of the heat treatment, is effective for the inactivation of *S. aureus* (Kim et al., 2013; Xie et al., 2017).

The concentration of nGO and GO were independent of the temperature reached during the irradiation of *S. aureus*-GO, *S. aureus*-nGO, *E. coli*-GO and *E. coli*-nGO the systems. This analytical data indicates that GO and nGO efficiently converted optical energy into thermal energy.

The heating efficiency of PTT is expressed as:

(1) $$e\,f\,f=\frac{T_{G\,O/n\,G\,O}}{T_{c\,o\,n\,t\,r\,o\,l}}$$

Where *T*_*GO/nGO*_ is the Temperature experience of the GO or nGO solution during photothermal irradiation, and *T*_*control*_ is one experience within the control group (Huang et al., 2008). As shown in Figure 9B, in the case of nGO, the heating efficiency of the PTT experiences cause a sudden increase to a dose of 20 J cm^–2^, after which it increases little until the maximum efficiency; no difference is observed in this behavior between 450 and 550 μg ml^–1^ concentrations. In the case of GO, it is observed that in the two concentrations used in this study there is an increase in efficiency up to around 40 J cm^–2^, after which the efficiency remains almost constant, giving a small increase up to 60 J cm^–2^. Figure 9C shows a table indicating the heating efficiency for doses used in this work.

The determination of the thermal efficiency shows that as a conversion of electromagnetic radiation into thermal energy, GO is superior to nGO. As the light dose increases the rate in which *eff* increases is larger for GO than for nGO, demonstrating the better capacity for converting energy.

In the same sense, this result corroborates with the overall behavior of GO in comparison with nGO in promoting microorganism reduction on the reported experiments in previous sections of this work.

### Graphene Oxide and Nanographene Oxide-Mediated Photothermal Therapy/Photodynamic Therapy in Fibroblasts

**FIGURE 10:**
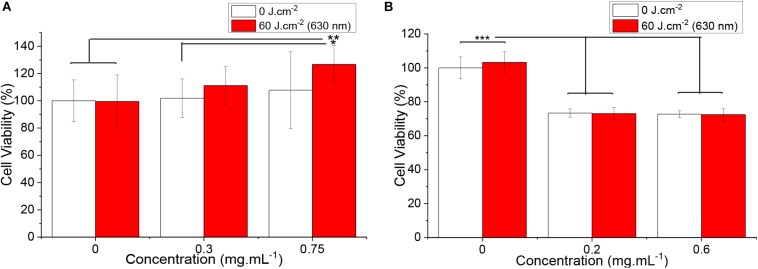
Human dermal fibroblasts viability when exposed to **(A)** GO and **(B)** nGO for 45 min and irradiated with 630 nm (60 J cm^–2^). White bars represent the groups that remained protected from light, while groups exposed to the light source are displayed in red. The ^∗^, ^∗∗^ and ^∗∗∗^ symbol the bars indicate statistically significant difference between the indicated groups according to Tuckey’s multiple comparisons (*p* ≤ 0.05, *p* ≤ 0.01, and *p* ≤ 0.001, respectively).

To assess the impact of PTT/PDT protocol using the most efficient parameters for *S. aureus*, inactivation with GO solutions, human neonatal dermal fibroblast cultures were incubated with 0.3 and 0.75 mg ml^–1^ solutions for 45 min and exposed to 60 J cm^–2^. No damage was observed and no statistically significant alteration to cell viability was promoted by the lowest concentration, even after the samples were irradiated (Figure 10). There was, however, an apparent increase in the number of viable cells when HDFn were exposed to 0.75 mg ml^–1^ GO solution and 60 J cm^–2^. To determine GO uptake and its influence on cell cycle, further studies must be conducted.

Nanographene oxide samples, however, presented a reduction of approximately 30% in cell viability for both concentrations 0.2 and 0.6 mg ml^–1^ in the presence/absence of light. These data suggest that the mild damage observed in the samples is independent of nGO concentration and light exposure, and may derive from nGO interaction with fibroblasts.

## Discussion

Liu et al. (2011) studied the antibacterial activity of GO and other carbon-based nanomaterials in experiments observing the effect of exposure time and concentration, the SEM images suggested that the bacteria exposed to GO are largely involved and almost uniformly enveloped by the GO sheets. Liu et al. (2012) obtained a similar result with the AFM images in the presence of large GO sheets and *E. coli* (GO size of 2–3 um and GO concentration 80 μg ml^–1^), they evaluated the antibacterial effect of different sizes of GO incubated for 2 h in *E. coli* solution. A total coating of the microorganism was observed. Large GO sheets more easily cover cells, resulting in cell viability loss. In contrast, small GO (10 nm) sheets adhere to the bacterial surface but cannot effectively isolate cells from the environment. According to Supplementary Figures S1A,C, the size of the GO and nGO sheets are around 2 μm and 340 nm, which allows both the *S. aureus* (around 1 μm) and *E. coli* (around 3 μm) to be fully covered with GO and nGO due the concentration of GO nGO used in this work (>300 μg ml^–1^).

The question now is why low concentrations of GO and nGO are required for the decontamination of *S. aureus* and high concentrations in the case of *E. coli*. The characteristics of the bacterial membrane and its interaction with GO were investigated by several authors. Akhavan and Ghaderi (2010) studies the antimicrobial effect of GO and rGO sheets, they found that Gram-negative *E. coli* is less susceptible than Gram-positive *S. aureus*. The Gram-positive species have thicker peptidoglycan layers compared to the Gram-negatives ones. These layers provide an additional protection by the outer membrane structures found in Gram-negative cells. Krishnamoorthy et al. (2012) reached to similar conclusion by comparing the GO susceptibility of Gram-positive *Steptococcus iniciae* and *E. coli*. Therefore, due to the greater susceptibility of *S. aureus*, it requires less concentration of GO and nGO to be disinfected and vice versa for the case of *E. coli* in the presence of GO and nGO.

The absence of cytotoxicity in Human Dermal Fibroblast neonatal cultures (HDFn), for PTT/PDT effects is in accordance with several studies suggesting the biocompatibility of GO. Chang et al. (2011) report that 0.2 mg ml^–1^ GO solutions presented mild cytotoxicity on pulmonary adenocarcinoma cells viability for longer incubation times such as 72 h. Bengtson et al. (2016) evaluated the cytotoxicity of GO solutions up to 0.2 mg ml^–1^ on murine lung epithelial cells for 24 h, finding no significant reduction on cell viability nor genotoxicity evidences. Yuan et al. (2011) observed a reduction on human hepatoma cells viability of 6% after 48 h and reported the influence of GO on cell cycle, displaying a minor reduction on the cells proliferation rate that was reflected on the alterations found in proliferation-related proteins. The reduced size of nGO may increase their uptake by the fibroblasts, allowing them to act directly on cell mechanisms, molecules and organelles, or disrupt the cell membrane via electrostatic interactions of the GO negative charges (derived from the oxygen groups) and the membrane positive charges (originated by phospholipids), as suggested by Liao et al. (2011). GO, however, may interact mainly with cell surface due its increased size, what has been previously reported by Chang et al. (2011). The negative influence on nGO on cell proliferation was reported by Cicuéndez et al. (2018) and may also explain the difference in the number of viable cells after 24 h following the PTT/PDT protocol: by delaying HDFn proliferation, fewer cells could be present when the MTT assay was conducted (Cicuéndez et al., 2018). The absence of further damage to the cells after sample irradiation was also reported by Tian et al. (2011), which verified no impact on nasopharyngeal epidermal carcinoma cells viability when incubated with 0.011 mg ml^–1^ nGO functionalized with polyethylene glycol (PEG) solutions for 24 h and exposed to 60 J cm^–2^ (Tian et al., 2011).

The results show that GO and nGO can sensitize the formation of _1_O^2^ and allow a temperature rise of 55°C to 60°C in together nGO and GO to exert combined PTT/PDT effect in the disinfection of gram-positive *S. aureus* and gram-negative *E. coli* bacteria used ultra-low doses (65 mW cm^–2^) of 630 nm light. Obtaining a better heating efficiency of PTT for GO y nGO between the light doses of 40 and 60 J cm^–2^. A complete elimination of *S. aureus* bacteria based on GO and nGO is obtained by using a dose of 43–47 J cm^–2^ for concentrations (high) of 300–750 μg ml^–1^ and 446.3–595 μg ml^–1^ for GO and nGO, respectively. In the case of *E. coli* bacteria, complete elimination is observed by also using a dose of 43–47 J cm^–2^ for concentrations of 1.13–1.5 mg ml^–1^ and 535.5–743.8 mg ml^–1^ for GO and nGO, respectively. In both cases to achieve a complete elimination of bacteria with low concentrations of GO and nGO a light dose of around 70 J cm^–2^ was used. The presence of high concentrations of GO allows the bacterial population of *S. aureus* and *E. coli* to be more sensitive to the use of PDT/PTT. The overlap area description shows the efficiency of *S. aureus* and *E. coli* bacteria disinfection in the presence of GO is similar to that of nGO. In human neonatal dermal fibroblast, HDFs, no significant alteration to cell viability was promoted by GO, even after the samples were irradiated, but in nGO is observed than a mild damage in the HDFs cells is independent of nGO concentration and light exposure.

In summary, we study GO and nGO for low dose red light actively bimodal photodynamic and photothermal therapeutic *in vitro* broad-spectrum antimicrobial. The applicability of graphene in many forms to act as microbial agent is a very promising topic. Graphene is a biocompatible specie and could find very good clinical applications for recent challenging situations concerning dermatologic infections.

## Data Availability Statement

All datasets generated for this study are included in the article/Supplementary Material.

## Author Contributions

MR carried out the synthesis, characterization, physicochemical and microbiological study, and wrote the manuscript. VM worked on the synthesis of graphene oxide and the manuscript review. CF was responsible for the threshold dose analysis and the manuscript review. IL worked on the cytological study in epithelial cells. CS and CM monitored the synthesis of graphene oxide and reviewed the manuscript. MP-D-S analyzed the morphology of the microbes by AFM. VB was involved in the discussion during the manuscript preparation and review, and discussion of the results. NI was involved in the discussion on the results and the manuscript.

## Conflict of Interest

The authors declare that the research was conducted in the absence of any commercial or financial relationships that could be construed as a potential conflict of interest.
