# Supplementary material for: Graphene Oxide Mediated Broad-Spectrum Antibacterial Based on Bimodal Action of Photodynamic and Photothermal Effects
Source: Front Microbiol. 2020 Jan 15;10:2995. doi: 10.3389/fmicb.2019.02995 (PMC6974586; doi:10.3389/fmicb.2019.02995)
Supplement: Supplementary file 1 [file Data_Sheet_1.PDF]

Supporting Information

Graphene Oxide mediated broad-spectrum antibacterial based on bimodal action of Photodynamic and Photothermal effects

María Paulina Romero[1, 2], Valeria Spolon Marangoni[3], Clara Gonçalves de Faria[1], Ilaiali Souza Leite[1], Cecília de Carvalho Castro e Silva[3], Camila Marchetti Maroneze[3], Marcelo A. Pereira-da-Silva [1], Vanderlei Salvador Bagnato[1], Natalia Mayumi Inada[1].

E1. Experimental process for bacterial suspension and treatment experiments

Stocks of E. coli (ATCC 8739) and S. aureus (ATCC 25923) strains were maintained in BHI (Brain-Heart infusion, Broth. Acumedia) supplement and glycerol (40%) at 4ºC. Prior to inoculation, the strains were transferred from the stock cultures (1 ml) to BHI supplement (9 ml) and incubated at 37ºC overnight. The cultures were used for preparation of bacterial suspension (10^8 Colony Forming Units/ml) in phosphate-buffer saline (PBS) and maintained at 6-7ºC for 2 hours. Subsequently, 2 mL of bacterial suspension were transferred to a 2 mL centrifuge tube for centrifugation (3000rpm, 10 min).

The resultant solutions were incubated for 45 min at 37ºC and protected from light. Afterwards, the different solutions (0.5 ml, approximately 10^8 CFU/ml) were placed in a 24-well plate and irradiated with red light (630 nm; 65.5 mW/cm^2 prototype “Biotable®”, developed by the Technological Support Laboratory, Sao Carlos Institute of Physics, University of Sao Paulo, São Carlos-SP, Brazil). Light doses used for the experiment were 20, 40, 60 and 80 J/cm^2). Lastly, the solutions were dissolved 5 times, transferred to the Petri dish (with solid BHI), and maintained for 24 h at 37ºC. Three replicates were performed for each experimental group.

E2. Experimental process in threshold dose analysis experiments

The dose-response curve, f(D), is a result of the cumulative effect on individuals as dose D increases. Therefore, the population fraction that dies at any energy D_0 is given by f(D) as the sum:

$$f(D_0) = \int_0^{D_0} g(D)dD$$

where g(D)dD represents the proportion of individuals with tolerances varying between D and D+dD, dD being an infinitesimal interval dose, and can be interpreted as the probability that one individual, randomly selected from the population, responds to stimulus D. The threshold dose distribution can be obtained by differentiating the experimental dose-response curve:

$$g(D_o) = {}^{df}\!\big/_{dD}\Big|_{Do}$$

The distribution can be characterized by an ΔD width ΔD, measured at half maximum (FWHM), and its peak center D_P, which corresponds to the maximal dose amplitude. De Faria et al. (2018) argued that D_P quantifies the characteristic resistance in the population, and ΔD is a measure of how heterogeneous the population is regarding the threshold dose. The variability is related to resistance.

To obtain dose-response curves, data was fitted to sigmoidal curves, threshold distributions were obtained by differentiating them and ΔD and D_P were calculated using the software Origin 8.

E3. Experimental process in vitro cellular uptake experiments

Human dermal fibroblast cells isolated from neonatal foreskin (HDFn cell line, catalog number C0045C, purchased from Thermo Fisher Scientific, Waltham, MA, USA) were cultured in DMEM (catalog number 460, Cultilab, Campinas, SP, Brazil) supplemented with 10 % (vv^{-1}) fetal bovine serum (FBS, catalog number 63, Cultilab) in a humidified incubator (37ºC, 5 % CO_2). For each experiment, 96-well plates were seeded with a suspension containing 5.10^4 cell/mL in DMEM supplemented with 10 % FBS. Cells were incubated for 45 minutes with GO (0.75 and 0.3 $mgmL^{-1}$) and nGo (0.6 and 0.2 $mgmL^{-1}$) solutions, prepared in phenol-free DMEM supplemented with 10 % FBS immediately before its use. Groups exposed to light were irradiated with a 630 nm LED device with a fluency of 60 Jcm^{-2} (corresponding to 15 minutes of exposure), while dark control groups remained protected from light. Cells were then washed with PBS twice before the media were replaced with phenol-free DMEM supplemented with 10 % FBS and incubated in humidified incubator for 24 hours.

Viability was evaluated indirectly with the MTT assay. Briefly, media were replaced with phenol-free DMEM supplemented with 10 % MTT stock solution (5 $mgmL^{-1}$, Sigma Aldrich, St. Louis, MO, USA) and incubated for 3 hours. Formazan crystals were dissolved with DMSO (Labsynth, Diadema, SP, Brazil) and the absorbance values were measured with a microplate reader (Multiskan Go, Thermo Fisher Scientific) at 570 nm. Cell viability was calculated considering the absorbance values of the group that wasn't exposed to GO/nGo nor submitted to 630 nm as 100 %. Data is expressed as the mean value ± standard deviation of three experiments with each group containing a triplicate (total n = 9). Statistical analysis was performed with GraphPad Prism 6.0, using the analysis of variance (ANOVA) followed by Tuckey's multiple comparisons.

Fig. S1 (a) and (d) GO and nGO sheet TEM . (b) and (e)TEM images of GO and nGO. (c) and (f) High resolution TEM images of GO and nGO and corresponding 2D FFT images.

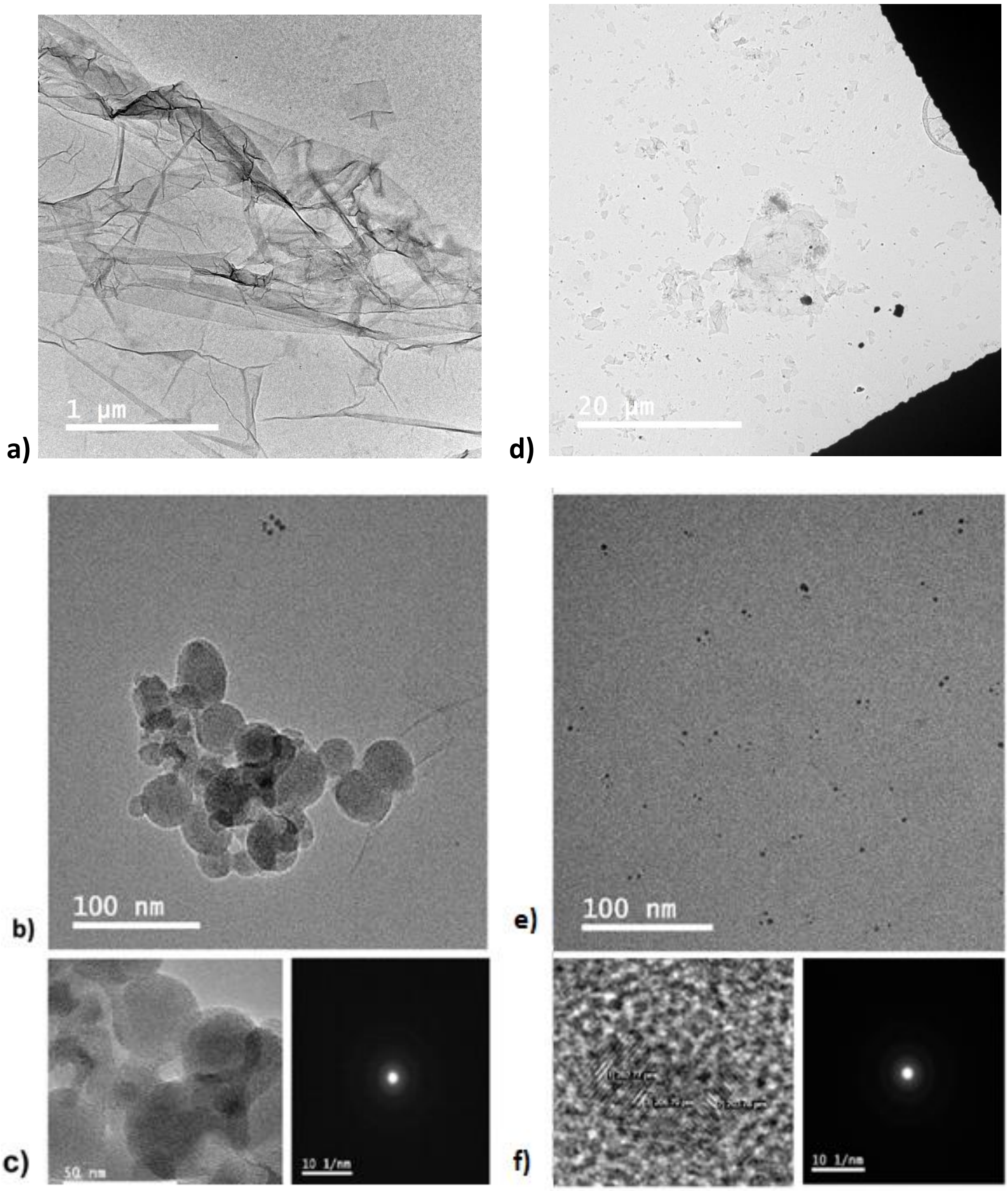

Figura .S2 FTIR spectrum data for GO and nGO

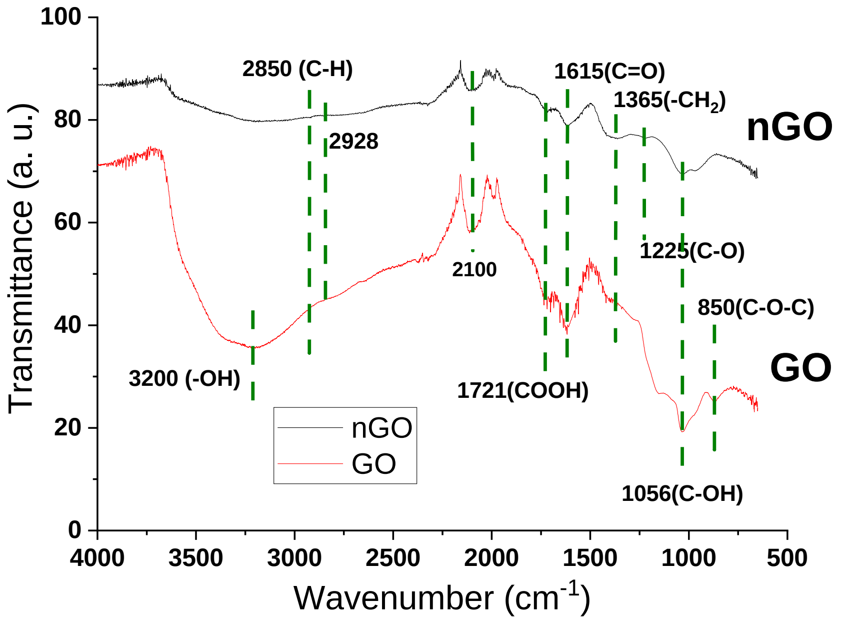

Figura S3. Raman spectrum for GO and nGO

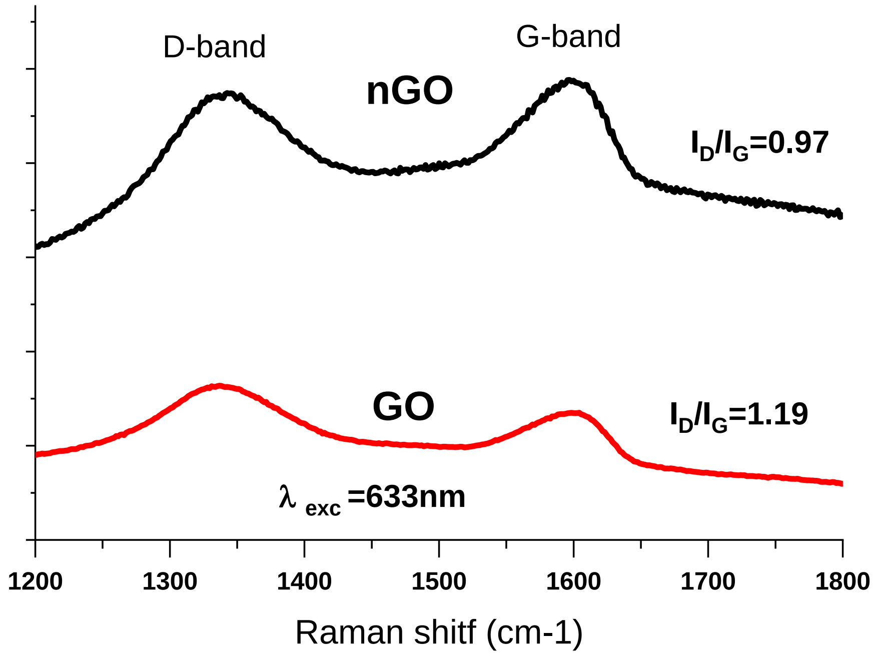

Fig. S4. DPBF Photoxidation from GO (GO + DPBF irradiated at different times 3, 6, 9, 12 and 15min). GO: 30ug ml^{-1}, DPBF: 5µM. Light irradiation: 630nm, 65.5 mW cm^{-2}.

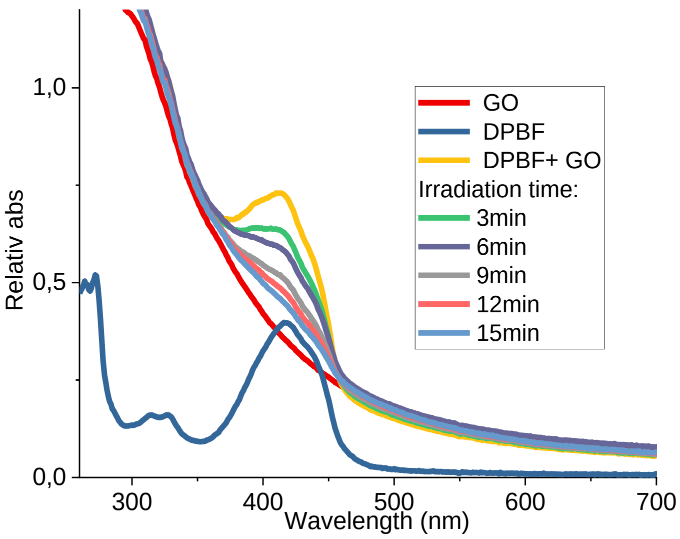

Fig. S5. Decay curve of DPBF from GO and nGO. DPBF = 5 µM.

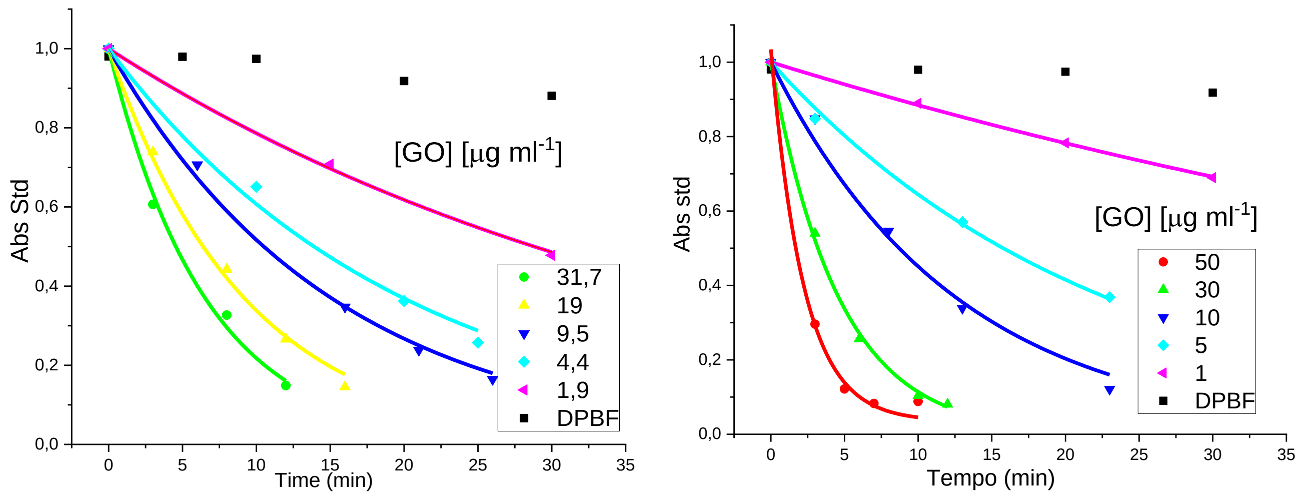

Fig. S6. Decay Tiime by photoxidation of GO + DPBF and nGO + DPBF. These times were obtained by fitting the S7 decay curves. 5 µm DPBF.

**GO** [µg ml^{-1}]	t_d [min] [Stand error]	**nGO** [µg ml^{-1}]	t_d [min] [Stand error]
1	81.51 ± 0.76	1.9	41.56 ± 0.93
5	22.76 ± 0.77	4.4	20.05 ± 1.04
10	12.56 ± 0.78	9.5	15.15 ± 0.45
30	4.60 ± 0.11	19.0	9.23 ± 0.30
50	2.21 ± 0.26	31.7	6.56 ± 0.30
